# Supplementary figures and images for: Population processes in cyber system variability
Source: PLoS One. 2022 Dec 27;17(12):e0279100. doi: 10.1371/journal.pone.0279100 (PMC9794046; doi:10.1371/journal.pone.0279100)

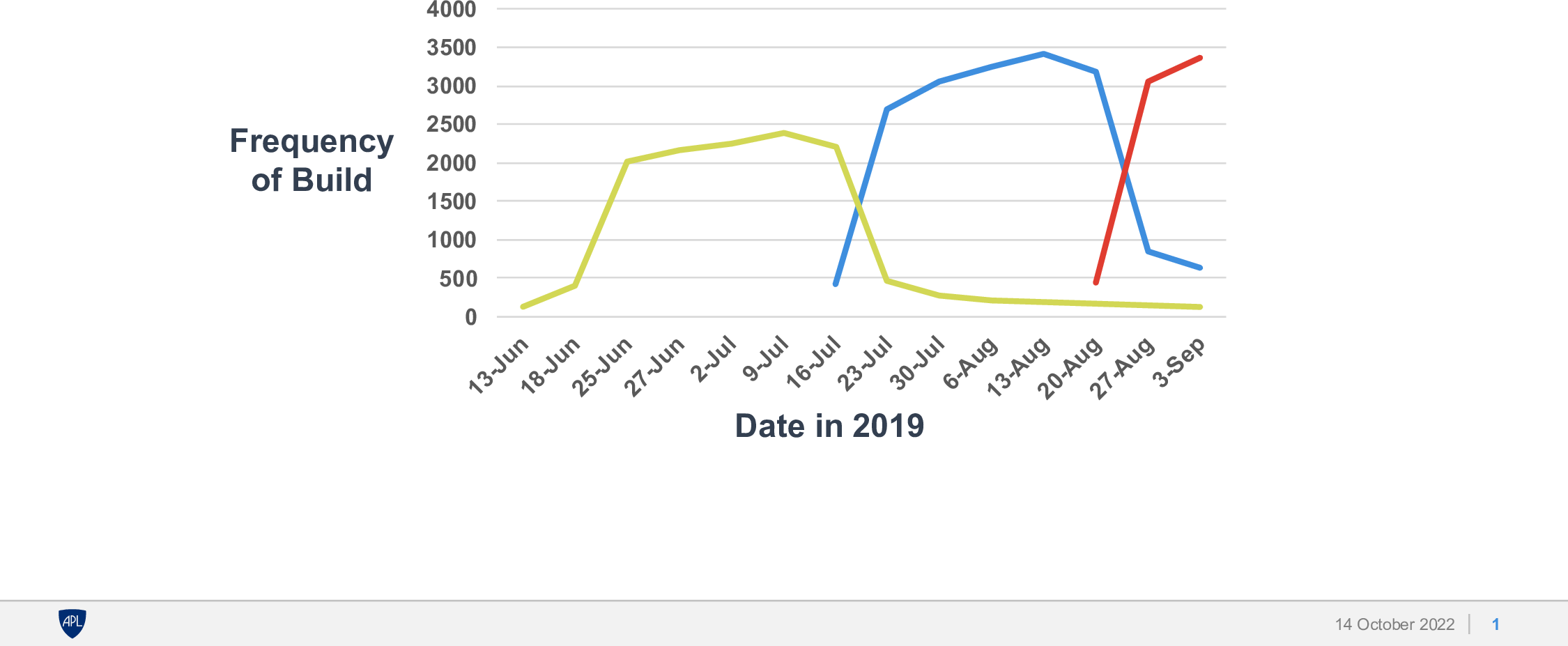

Supplement: S1 Fig — The colors represent different updates of the build (see text for explanation): 17763.615 (light blue), 17763.557 (yellow), 17763.678 (red), 17763.529 (dark blue), and 17763.503 (gold). (TIF) [file pone.0279100.s001.tif]
