# Supplementary material for: Population processes in cyber system variability
Source: PLoS One. 2022 Dec 27;17(12):e0279100. doi: 10.1371/journal.pone.0279100 (PMC9794046; doi:10.1371/journal.pone.0279100)
Supplement: S1 File — Codes that implement the methods [44, 45]. (PDF) [file pone.0279100.s004.pdf]

Supporting information

S1 Appendix. Details of the data shown in Fig 2A.

In 2019–2020, we collected data on a network of 7000 computers using the Microsoft operating system. Fig 2A reflects the dynamics of OS updates for a subset of about 335 computers; in this section we will explain this choice.

Microsoft Windows software has three labels that characterize the current OS version: release, build, and update. Microsoft makes about two releases of Windows 10 per year, with a nomenclature that has evolved over the past five years; we characterize it as YYMM. Within the release there are specific builds, labeled YYbbb, where bbb is a three-digit number. Most of these are internal to Microsoft, so externally we assume only one build for each release. Every month or so there are updates to the build, labeled YYbbb.uuuu. In S1 Fig, we show the distribution of build 17763 during the first quarter of the period (16 July 2019–3 March 2020) in which we collected data.

We sampled from the network of 7000 computers, using these criteria to include data from a computer or not: i) the computer appeared in the dataset every week from July 2019 to March 2020, ii) the computer was among the 424 that transitioned to build 17763.615 on 16 July 2019, and iii) the computer did not revert to a previous build when it made a transition. These criteria resulted in a dataset of 334 computer operating systems.

Using these criteria allowed us to focus on the update dynamics within a single build, to constrain our definition of transition to update transitions, and to exclude build-build transitions. Because we focused on computers that transitioned to build 17763.615 on 16 July 2019, we knew that all of the tracked computers started at the same OS. Doing so kept our longitudinal sample set well characterized in terms of update transitions but excluded most of the computers in the larger network.

We show these data in Table A and S1 Fig and their summary (leading to Fig 2A) in Table B.

S1 Fig. The distribution of updates to the Microsoft Windows 10 build 17763 in the network of 7000 computers that we sampled during the first quarter of our data collection. The colors represent different updates of the build

(see text for explanation): 17763.615 (light blue), 17763.557 (yellow), 17763.678 (red), 17763.529 (dark blue), and 17763.503 (gold).

S2 Appendix. Connecting continuous and discrete time models.

To demonstrate the nature of the continuous time versions of Eqs 3–5, we replace transition probabilities by transition rates. That is, we let λ_{ud} and λ_{du} denote the rates of the F $\rightarrow$ NF and NF $\rightarrow$ F transitions, respectively, in the sense that the probabilities of the F $\rightarrow$ NF and NF $\rightarrow$ F transitions in the next small interval of time Δt are $\lambda_{ud}\Delta t + o(\Delta t)$ and $\lambda_{du}\Delta t + o(\Delta t)$, respectively, where $o(\Delta t)$ corresponds to terms that are higher order in Δt. As noted in the main text, we can formally connect the rates and probabilities of transition by $p_{ud} = 1 - e^{-\lambda_{ud}}$ and $p_{du} = 1 - e^{-\lambda_{du}}$.

The logic relating the probability of a component being functional over time remains the same. That is, for a component to be functional at time $t + \Delta t$, it either had to be functional at time t and remain that way during the next Δt units of time or had to be nonfunctional at time t and transition to functional in the next Δt units of time. Thus, the continuous time analogue of Eq 3 is

$$P_F(t + \Delta t) = P_F(t)(1 - \lambda_{ud}\Delta t - o(\Delta t)) + P_{NF}(t)\lambda_{du}(\Delta t + o(\Delta t)). \tag{19}$$

We now Taylor expand both sides in powers of Δt to obtain

$$P_F(t) + \frac{dP_F}{dt}\Delta t = P_F(t) - P_F(t)\lambda_{ud}\Delta t + P_{NF}(t)\lambda_{du}\Delta t + o(\Delta t). \tag{20}$$

We subtract $P_F(t)$ from both sides, divide by Δt, let $\Delta t \rightarrow 0$, and use the property that $o(\Delta t)/\Delta t \rightarrow 0$ as $\Delta t \rightarrow 0$ to obtain

$$\frac{dP_F}{dt} = -P_F\lambda_{ud} + P_{NF}\lambda_{du}. \tag{21}$$

This equation shows how the probability that a component is functional "flows" out of the functional state at rate λ_{ud} and into the functional state from the nonfunctional state at rate λ_{du}.

Similar reasoning leads to the equation for $P_{NF}(t)$,

$$\frac{dP_{NF}}{dt} = -P_{NF}\lambda_{du} + P_F\lambda_{ud}. \tag{22}$$

The steady states of Eqs 21 and 22 give a relationship between the steady state

probabilities, i.e.,

$$\overline{P}_{NF} = \frac{\lambda_{ud}}{\lambda_{du}} \cdot \overline{P}_F. \tag{23}$$

However, it must always be true that the probabilities of being in either the functional or nonfunctional state sum to 1. Setting $\overline{P}_{NF} + \overline{P}_F = 1$, using Eq 23, and simplifying, we obtain

$$\overline{P}_F = \frac{\lambda_{ud}}{\lambda_{du} + \lambda_{ud}}, \tag{24}$$

which is the continuous time analogue of Eq 5.

Clearly, reaching Eq 24 requires more mathematical chops than reaching Eq 5, which is why we focused on discrete time models. But there is also an advantage. We can convert Eq 21 to an equation for P_F only by writing $P_{NF} = 1 - P_F$ and solve the resulting equation by the method of an integrating factor. The resulting solution with the initial condition $P_F(0) = 1$, with $\lambda_T = \lambda_{ud} + \lambda_{du}$, is

$$P_F(t) = \frac{\lambda_{du}}{\lambda_T} + \frac{\lambda_{ud}}{\lambda_T} e^{-\lambda_T t}. \tag{25}$$

The probability that the component is nonfunctional is, of course, 1 minus the right-hand side of Eq 25, which can also be obtained by following an analogous approach for Eq 22. Thus, we have demonstrated that the decay toward the steady state is determined by the total rate of transitions; this was observed in the results shown in Fig 10.

S3 Appendix. The Gaussian approximation for the forward equation.

To ensure that this analysis is self-contained, we first derive the rules of total expectation and total variance, then apply them to the Gaussian approximation.

The rules of total expectation and total variance

In this section, we illustrate the rules [7, 44] for discrete random variables. In particular, if X and Y are random variables, $p(x)$ is the probability that $X = x$ and $p(x|y)$ is the probability of $X = x$ given that $Y = y$ then from the definition of conditional probability,

$$p(x) = \Sigma_y p(x|y). \tag{26}$$

It then directly follows [45] that the expected value of X is

$$\mathcal{E}(X) = \mathcal{E}_Y[\mathcal{E}(X|Y)]. \tag{27}$$

By definition, the variance of X is $Var(X) = \mathcal{E}(X^2) - \mathcal{E}(X)^2$ so that if we condition on Y and use Eq 27, we have

$$Var(X) = \mathcal{E}_Y[\mathcal{E}(X^2|Y)] - \{\mathcal{E}_Y[\mathcal{E}(X|Y)]\}^2. \tag{28}$$

Using the definition of variance, we rewrite this equation as

$$Var(X) = \mathcal{E}_Y\{[Var(X|Y)] + \mathcal{E}(X|Y)^2\} - \{\mathcal{E}_Y[\mathcal{E}(X|Y)]\}^2 \tag{29}$$

so that

$$Var(X) = \mathcal{E}_Y[Var(X|Y)] + \left(\mathcal{E}_Y[\mathcal{E}(X|Y)^2] - \{\mathcal{E}_Y[\mathcal{E}(X|Y)]\}^2\right). \tag{30}$$

We recognize that the term in ( ) on the right-hand side of Eq 30 is the variance of $\mathcal{E}(X|Y)$ so that we conclude

$$Var(X) = \mathcal{E}_Y[Var(X|Y)] + Var_Y[\mathcal{E}(X|Y)]. \tag{31}$$

Application to the Gaussian approximation of the forward equation

To simplify notation, we drop the tildes and subscripts in Eq 9 and let $X(t) \sim \mathcal{B}(N_0 - N(t), p_{du})$ and $Y(t) \sim \mathcal{B}(N(t), p_{ud})$ to rewrite Eq 9 as

$$N(t+1) = N(t) + X(t) - Y(t), \tag{32}$$

where $X(t)$ and $Y(t)$ are independent binomially distributed random variables. Their means are, respectively, $p_{du}(N_0 - N(t))$ and $p_{ud}N(t)$.

We let $m(t)$ denote the mean value of $N(t)$; this is evaluated directly from the dynamics in Eq 9 using the rule of conditional expectation

$$\begin{aligned} m(t+1) = \mathcal{E}(N(t+1)) &= \mathcal{E}_{N(t)}[\mathcal{E}(N(t+1)|N(t))] \\ &= \mathcal{E}_{N(t)}[N(t) + p_{du}(N_0 - N(t)) - p_{ud}N(t)]. \end{aligned} \tag{33}$$

Combining terms in Eq 33, we conclude that

$$\begin{aligned} m(t+1) &= p_{du}N_0 + (1 - p_{du} - p_{ud})m(t) \\ &= m(t) + p_{du}N_0 - (p_{du} + p_{ud})m(t). \end{aligned} \tag{34}$$

The steady state value of the mean number of functional cyber components, $\overline{m}$, obtained by setting $m(t+1) = m(t) = m_s$ is

$$m_s = N_0 \cdot \frac{p_{du}}{p_{ud} + p_{du}}. \tag{35}$$

According to Eq 31, the variance of $N(t+1)$ is

$$Var(N(t+1)) = \mathcal{E}_{N(t)}[Var(N(t+1)|N(t))] + Var[\mathcal{E}_{N(t)}(N(t+1)|N(t))]. \tag{36}$$

To evaluate the first term on the right-hand side of Eq 31, we compute

$$Var(N(t+1)|N(t)) = Var(X(t)|N(t)) + Var(Y(t)|N(t)) - 2Cov(X(t), Y(t)|N(t)), \tag{37}$$

where $Cov(X(t), Y(t)|N(t))$ is the covariance of $X(t)$ and $Y(t)$, which in this case is zero because they are independent random variables. Furthermore, since $X(t)$ and $Y(t)$ both have binomial distributions,

$$Var(N(t+1)|N(t)) = (N_0 - N(t))p_{du}(1-p_{du}) + N(t)p_{ud}(1-p_{ud}). \tag{38}$$

Hence the first term on the right-hand side of Eq 36 is

$$\mathcal{E}_{N(t)}[Var(N(t+1)|N(t))] = (N_0 - m(t))p_{du}(1-p_{du}) + m(t)p_{ud}(1-p_{ud}). \tag{39}$$

The second term on the right-hand side of Eq 36 is

$$\begin{aligned} Var[\mathcal{E}_{N(t)}(N(t+1)|N(t))] &= Var[N(t) + (N_0 - N(t))p_{du} - p_{ud}N(t)] \\ &= Var[p_{du}N_0 + (1 - p_{du} - p_{ud})N(t)] \\ &= (1 - p_{du} - p_{ud})^2 Var(N(t)), \end{aligned} \tag{40}$$

where we have used $Var(cX) = c^2 Var(X)$ for any random variable X and constant c.

We combine Eqs 36–40 to obtain

$$v(t+1) = (N_0 - m(t))p_{du}(1-p_{du}) + m(t)p_{ud}(1-p_{ud}) + (1 - p_{du} - p_{ud})^2 v(t). \tag{41}$$

Setting $v(t+1) = v(t) = v_s$ in Eq 41, we obtain

$$v_s = \frac{(N_0 - m_s)p_{du}(1-p_{du}) + m_s p_{ud}(1-p_{ud})}{1 - (1 - p_{du} - p_{ud})^2}. \tag{42}$$

Eqs 35 and 42 generalize to any distribution for which the mean and variance of the transition process can be explicitly computed. For example, the beta binomial distribution in the Discussion could be used as a direct generalization, but that is beyond the scope of this paper.

S4 Appendix. Codes that implement the methods. In this section, we provide the codes to i) generate the data in Fig 2A, ii) generate the results for the two-state model, and iii) generate the results for the model with multiple operating systems.

Code to generate the data in Fig 2A

```
#wrangling the OS data
Most_recent=seq(from=1, to=10, length=10)
Current_version=array(0,c(10,10))
Current_version[1,1]=324
Current_version[2,2]=309
Current_version[3,2]=3
Current_version[3,3]=281
Current_version[4,3]=7
Current_version[4,4]=267
Current_version[5,3]=1
Current_version[5,4]=28
Current_version[5,5]=11
Current_version[6,4]=5
Current_version[6,5]=3
Current_version[6,6]=307
Current_version[7,6]=3
Current_version[7,7]=298
Current_version[8,7]=4
Current_version[8,8]=306
Current_version[9,8]=3
Current_version[9,9]=310
Current_version[10,9]=1
Current_version[10,10]=296


All_machines=rep(0,10)
for(os in 1:10)
{
  All_machines[os]=sum(Current_version[os,])
}
```

```
freq_current=array(0,c(10,10))


for(os in 1:10)
{
 for(mc in 1:10)
 {
   freq_current[os,mc]=Current_version[os,mc]/All_machines[os]
 }
}
#look at the data
plot(Most_recent,Most_recent,type="l",lwd=1,lty=3,
ylab="Current␣OS",xlab="Most␣recent␣OS")
for(os in 1:10)
{
  for(mc in 1:10)
  {
    if(Current_version[os,mc]>0)
    {
      points(os,mc,pch=16)
      #cex=4*freq_os[OS[n,t_shift[i_s]],t_shift[i_s]]
    }
  }
}
plot(Most_recent,Most_recent,type="l",lwd=1,lty=3,
ylab="Current␣OS",xlab="Most␣recent␣OS")
for(os in 1:10)
{
  for(mc in 1:10)
  {
    if(Current_version[os,mc]>0)
    {
      points(os,mc,cex=log10(Current_version[os,mc]),pch=16)
      #cex=4*freq_os[OS[n,t_shift[i_s]],t_shift[i_s]]
    }
```

```
  }
}
plot(Most_recent,Most_recent,type="l",lwd=1,lty=3,
ylab="Current␣OS",xlab="Most␣recent␣OS")

for(os in 1:10)
{
  for(mc in 1:10)
  {
    if(Current_version[os,mc]>0)
    {
      points(os,mc,pch=16)
      #cex=4*freq_os[OS[n,t_shift[i_s]],t_shift[i_s]]
    }
  }
}
plot(Most_recent,Most_recent,type="l",lwd=1,lty=3,
ylab="Current␣OS",xlab="Most␣recent␣OS")

for(os in 1:10)
{
  for(mc in 1:10)
  {
    if(Current_version[os,mc]>0)
    {
      points(os,mc,cex=log10(Current_version[os,mc]),pch=16)
      #cex=4*freq_os[OS[n,t_shift[i_s]],t_shift[i_s]]
    }
  }
}
```

Code to generate results for the model with two states

```
#This is Mangel and Brown 2 State System


library(fields)
require(graphics)



#First make parameters


N_0=100 #number of cyber components ins
p_ud=0.08
p_du=0.2
p_bar=p_du/(p_du+p_ud)

n_50=c(20,20,20,20)
sigma_n=c(10,10,10,10)
n_50_prime=c(70,60,50,40)
sigma_n_prime=c(10,10,10,10)

#*************  Make the Performance Function ************
I_n=N_0+1
n=seq(from=0,to=N_0,length=I_n)

#Unlike Mangel and McEver (2021), components only up or down so that
phi=array(0,c(4,I_n))
for(i_c in 1:4)
{
 for(i_n in 1:I_n)
 {
   term_u = exp( (n_50[i_c]-n[i_n])/sigma_n[i_c])
   phi_u=1/(1+term_u)

   term_d = exp((N_0-n[i_n]-n_50_prime[i_c])/sigma_n_prime[i_c])
   phi_d=1/(1+term_d)
```

```
   phi[i_c,i_n]=phi_u*phi_d
 }#end of loop over components
}#end of loop over cases



plot(n,phi[1,],type="l",lwd=3,xlab="Number␣of␣functional␣components",
 ylab="Performance")
for(i_c in 2:4)
{
  lines(n,phi[i_c,],lwd=3)
}


#*************** Simulate Some Trajectories ******************

#addtional parameters
T= 100
time=seq(from=1, to = T, length=T)

K=100 #number of simulations of performance

N=array(0,c(K,T))
Mean_n=rep(0,T)
N[,1]=N_0

for(t in 1:(T-1))
{

  for(k in 1:K)
  {
    delta_N_plus=rbinom(1,N_0-N[k,t],p_du)
    delta_N_minus=rbinom(1,N[k,t],p_ud)
```

```
    #
    #
    N[k,(t+1)]=N[k,t]+delta_N_plus-delta_N_minus
  }#end of state loop
}#end of time loop

plot(time,N[1,],type="l",lwd=3,col=2,ylim=c(40,N_0),xlab="Time",
ylab="Number␣of␣functional␣components")
for(k in 2:20)
{
  lines(time,N[k,],lwd=2,col=k+1)
}

for(t in 1:T)
{
  Mean_n[t]=mean(N[,t])
}
lines(time,Mean_n,lwd=5)


#now make the distribution of performance
MR=array(0,c(K,T))

n_50=20
sigma_n=10
n_50_prime=40
sigma_n_prime=10

for(k in 1:K)
{
  for(t in 1:T)
  {
  term_u = exp( (n_50-N[k,t])/sigma_n)
  phi_u=1/(1+term_u)
```

```
  term_d = exp((N_0-N[k,t]-n_50_prime)/sigma_n_prime)
  phi_d=1/(1+term_d)

  MR[k,t]=phi_u*phi_d

  }
}

quartz()
par(mfrow=c(2,2))
hist(MR[,5],xlab="Performance",ylab="Frequency",main="t=5",
xlim=c(0.5,1))
hist(MR[,30],xlab="Performance",ylab="Frequency",main="t=30",
xlim=c(0.5,1))
hist(MR[,70],xlab="Performance",ylab="Frequency",main="t=70",
xlim=c(0.5,1))
hist(MR[,90],xlab="Performance",ylab="Frequency",main="t=90",
xlim=c(0.5,1))

#********************** Solve the forward equation *****
#Reset the parameters
N_0=100 #number of cyber components ins
p_ud=0.08
p_du=0.2  #look at time 20

p_du=0.1  #look at time 40

p_du=0.05 #time 60

p_d_s_1=toString(p_ud)
p_d_s_2=toString(p_du)

leg=cbind("p_ud,␣p_du=",p_d_s_1,p_d_s_2)
```

```
#
#quartz()
par(mfrow=c(1,1))
F=array(0,c(N_0+1,T)) #true density (forward equation)
F_gau=rep(0,N_0+1)

#set initial condition: delta function
for(i_n in (N_0):(N_0))
{
  F[i_n,1]=1
} #end of loop over n

#uncomment here for a broader range of initial conditions
for(i_n in (N_0-31):(N_0))
{
  #F[i_n,1]=1/32
} #end of loop over n




N_1=N_0+1
n=seq(from=0,to=N_0,length=N_1)
m=seq(from=0,to=N_0,length=N_1)
#set the identity matrix
#I[m,j,k,n]=1 if n=m+j-k, 0 otherwise
I=array(0,c(N_1,N_1,N_1,N_1))
for(i_n in 1:N_1)
{
  for(i_m in 1:N_1)
  {
    for(j in 0:N_0-m[i_m])
    {
```

```
      for(k in 0:m[i_m])
      {
        if( m[i_m]+j-k == n[i_n])
        {#print("identity")
          I[i_m,j,k,i_n]=1}
      }
    }
  }
}




#loop over  time, then m and n

#for(t in 1:(T-1))
  for(t in 1:60)
{
  #datum=cbind("Doing t = ",t)
  #print(datum) #this piece of the code takes time, so
  #that time is printed to let us know where we are
  for(i_n in 1:N_1)
  {
    F[i_n,(t+1)]=0

    for(i_m in 1:N_1)
    {

      for(j in 0:N_0-m[i_m])
      {
        for(k in 0:m[i_m])
        {
          if(m[i_m]+j-k == n[i_n])
          {
```

```
          F[i_n,(t+1)]=F[i_n,(t+1)]+F[i_m,t]*
          dbinom(j,N_0-m[i_m],p_du,log=FALSE)*dbinom(k,m[i_m],
          p_ud,log=FALSE)

        }

      } #end of k loop
    }#end of j loop
  }#end of m loop
} #end of loop over n


} #end of loop over t




#look at the densities
#quartz()
plot(n,F[,1],type="l",lwd=4,xlab="Uncompromised␣units,␣n",
ylab="Density",ylim=c(0,0.2),main=leg)
for(t in 2:T)
{
  lines(n,F[,t],type="l",lwd=4,col=t)
}

#******* Now compare the Gaussian Approximation and
# Solution of Forward Eqn *******
m_s=N_0*p_du/(p_ud+p_du) #Eqn 17

#Numerator and demoninator for v_s (#Eqn 25)
numerator=(N_0-m_s)*p_du*(1-p_du)+m_s*p_ud*(1-p_ud)
denominator=1-(1-p_du-p_ud)^2
v_s=numerator/denominator
```

```
for(i_n in 1:N_1)
{
  F_gau[i_n]=exp( -(n[i_n]-m_s)^2/(2*v_s))
}

F_gau=F_gau/sum(F_gau)



plot(n,F[,60],type="l",lwd=4,xlab="Uncompromised␣units,␣n",
ylab="Density",ylim=c(0,0.1),col="gray",main=leg)
lines(n,F_gau,col=4,lwd=3,lty=3)
```

Code to generate the results for the model with multiple operating systems

```
#This is tge code for the model with multiple operating systems (OSs)

#first step is the sequence of operating systems

T=300
#set the operating systems
K=rep(1,T)
t_shift=rep(0,19)

for(t in 1:8 )
{K[t]=1}
t_1=8
t_shift[1]=8

for(t in 9:16 )
{K[t]=2}
t_2=16
t_shift[2]=16

for(t in 17:25 )
{K[t]=3}
t_3=25
t_shift[3]=25

for(t in 26:35 )
{K[t]=4}
t_4=35
t_shift[4]=35

for(t in 36:45)
{K[t]=5}
t_5=45
```

```
t_shift[5]=45

for(t in 46:56 )
{K[t]=6}
t_shift[6]=56

for(t in 57:70)
{K[t]=7}
t_shift[7]=70

for(t in 71:90 )
{K[t]=8}
t_shift[8]=90

for(t in 91:101 )
{K[t]=9}
t_shift[9]=101

for(t in 102:113 )
{K[t]=10}
t_shift[10]=113

for(t in 114:124 )
{K[t]=11}
t_shift[11]=124

for(t in 125:140 )
{K[t]=12}
t_shift[12]=140

for(t in 141:180 )
{K[t]=13}
t_shift[13]=180
```

```
for(t in 181:200 )
{K[t]=14}
t_shift[14]=200

for(t in 201:220 )
{K[t]=15}
t_shift[15]=220

for(t in 221:250 )
{K[t]=16}
t_shift[16]=250

for(t in 251:275 )
{K[t]=17}
t_shift[17]=275

for(t in 276:280 )
{K[t]=18}
t_shift[18]=280

for(t in 281:T )
{K[t]=19}
t_shift[19]=T


plot(K,xlab="Time,␣t",ylab="Most␣Recent␣OS")



#next make the transition probability function,
#given that a transition occurs
f=array(0,c(K[T],K[T],T)) #density function

#This is the probability of transition from OS(t)=l to OS(t+1)=K
#depends upon k,l, and t
```

```
#maximum value of l is K[t]-1 since, this density is
# based on the assumption
#that a transition occurs. Note we are assuming
# that if an update occurs
# between time t and t+1, so that K[t+1]=K[t]+1
#cannot transition to the
#new system (a problem of a discrete time model).
#  This  requires renormalization
#of f(k,l,t+1), to account for not being able to be at K[t+1].



for(t in (t_1+1):(T-1))
{
    for(l in 1: (K[t]-1))
    {
     for(k in (l+1):(K[t]))
     {
mu=0.5*(K[t]+l) #mean of the transition function is the average of
#current OS and maximum could transition to

sigma=0.2*mu #this makes coefficient of variation 20\% but also state
#independent

#sigma=1.5  #this would make coefficient of variation state dependent

f[l,k,t] =exp(-(k-mu)^2/(2*sigma^2)) #Gaussian transition density
     }#end of the k loop
    }#end of l loop
}#end of time loop




#renormalize so that they sum to over k and t
```

```
for(t in (t_1+1):(T-1))
{
  for(l in 1: (K[t]-1))
  {
   f[l,,t] =f[l,,t]/sum(f[l,,t])
  }
}


t_plot=190
l_start=1
plot(f[l_start,,t_plot],type="l",lwd=4,ylim=c(0,.5),
xlab="OS at time t",ylab="Probability of transition",
main="Transitions at t=190")
for(i_t in 1:5)
{
  lines(f[(l_start+2*i_t),,t_plot],type="l",lwd=4,col=i_t+1)
}
legend(3, 0.5, legend=c("OS(t)=1", "OS(t)=3","OS(t)=5","OS(t)=7",
"OS(t)=9","OS(t)=11"),
       col=c(1,2,3,4,5,6), lty=1, lwd=4, cex=0.4)
abline(v=K[190],lwd=3,lty=3)



t_plot=290
l_start=1
plot(f[l_start,,t_plot],type="l",lwd=4,ylim=c(0,.3),
xlab="OS at time t",ylab="Probability of transition",
main="Transitions at t=290")
for(i_t in 1:5)
{
  lines(f[(l_start+2*i_t),,t_plot],type="l",lwd=4,col=i_t+1)
}
legend(3, 0.25, legend=
c("OS(t)=1", "OS(t)=3",
```

```
 "OS(t)=5","OS(t)=7","OS(t)=9","OS(t)=11"),
        col=c(1,2,3,4,5,6), lty=1, lwd=4, cex=0.4)
 abline(v=K[290],lwd=3,lty=3)

 #********** Structural set up done ****************

 #first simulate to build intuition

 theta=0.025 #base case, other two values are
# theta=0.05
# theta=0.1
 #To duplicate Fig 11, one needs to run this
 # part of the code three times for the three values of theta
 p_trans=1-exp(-theta)

 #Simulate OS systems
 N=1000

 #n = index for individual computer

 OS=array(0,c(N,T))
 OS[,1]=1

  for(n in 1:N)
  {
    #first loop over machines, then over time
    for(t in 1:(T-1))
    {

      Z=runif(1,0,1)
      #print(Z)
      if(Z>=p_trans)
     {
        #nothing happens
```

```
    OS[n,t+1]=OS[n,t]
  }

  #cannot update if at the most current system
  if(OS[n,t]==K[t])
  {
    OS[n,t+1]=OS[n,t]
  }

  if(Z<p_trans & OS[n,t]<K[t])
  {
    #something happens

    Z1=runif(1,0,1)

    g9=1
    sum_check=f[OS[n,t],OS[n,t]+1,t]
    k=OS[n,t]+1
     if(Z1 <= sum_check)
     {
       OS[n,t+1]=k
       g9=-1
     }


    while(g9>0)
    {
      if(Z1 <= sum_check)
      {
        OS[n,t+1]=k
        g9=-1
      }

      k=min(k+1,K[t])
```

```
          sum_check=sum_check+f[OS[n,t],k,t]
        }


      }#end of something happens


    }#end of loop over time
    } #end of loop over machines



# make the frequency distribution of OS in time
  freq_os=array(0,c(K[T],T))
  #
    for(t in 1:T)
    {
      for(n in 1:N)
      {
        for(k in 1:K[t])
        {
          if(OS[n,t]==k)
          {
            freq_os[k,t] =freq_os[k,t]+1
          }
        }
      }
    }

  #renormalize
  freq_os=freq_os/N



  #try to make a plot like Alan sent on 13 March.

  theta_string=toString(theta)
  leg=cbind("Theta␣=",theta_string)
```

```
#set the axes
plot(K[t_shift[2]],K[t_shift[2]],main=leg,type="p",xlim=c(1,20),
ylim=c(1,20),pch=15,xlab="Max␣OS",ylab="Observed␣OS")
for(i_s in 3:19)
{
  points(K[t_shift[i_s]],K[t_shift[i_s]],pch=15)
}

for(n in 1:1000)
{
  for(i_s in 3:19)
  {
points(K[t_shift[i_s]],OS[n,t_shift[i_s]],col=1,
cex=3*freq_os[OS[n,t_shift[i_s]],t_shift[i_s]])
  }
}

#*******  ready for the forward equation **************

p=array(0,c(K[T],T))

#initial condition
p[1,1]=1

plot(p[,1],xlab="Operating␣System",ylab="Pr{OS(1)}")

#remain at OS(t)=1 if there is no transition by time t
for(t in 2:T)
{
  p[1,t]=exp(-theta*(t-1))
}

#now can really do something!
#nothing happens before K[T]>=2
```

```
  for(t in (t_1+1):295)
  {
    #want to know the probability of being at OS k at time t+1.
    #so k ranges from 2 to K(t)
    #and l ranges from 1 to K(t)-1

  #if(K[t+1]==K[t])
 # {
    for(k in 2:K[t])
      {

      #either already at that state and no transition
      p[k,t+1]=p[k,t]*exp(-theta)

     # or at l=1,2,...k-1 and transition from there
      #now ready for the sum over transition possibilities
      sum_trans=0
      for(l in 1:(k-1))
      {
       sum_trans=sum_trans + p[l,t]*f[l,k,t]
      }#end of loop over l
      p[k,t+1]=p[k,t+1]+sum_trans*p_trans
    }#end of loop over k
#  } #end of case when there is no jump between t and t+1






    #as explained above (because of the
    #possiblity of a new OS between t and t+1)
    #renormalize
    p[,t+1]=p[,t+1]/sum(p[,t+1])
```

```
  }# end of loop over time



  #********** Now get vulnerabilities ************


  #Want p_exp(k,t)=Pr{OS k is exploited at time t}


  p_exp=array(0,c(K[T],T))


  #exploitation rate goes down with release (get better at protected)


  lam_exp=rep(0,K[T])
  #OS=1 is 95% exploited at t=100
  lam_exp_base=-log(0.05)/100

for(k in 1:K[T])
{
  lam_exp[k]=lam_exp_base/(1+ .05*k)
}

plot(lam_exp,type="l",lwd=4,xlab="Operating␣System",
ylab="Rate␣of␣exploitation")

#OS 1 vulnerable from the outset
for(t in 1:T)
{
  p_exp[1,t]=1 - exp(-lam_exp[1]*(t-1))


}


 #now the other systems


for(n in 2:18)
{
```

```
t_start=t_shift[n-1]+1
print(t_start)
  for(t in t_start:T)
  {
  p_exp[n,t] =1 - exp(-lam_exp[n]*(t-t_start))
  }
}




plot(p_exp[1,],type="l",lwd=2,xlab="Time,␣t",ylab="Pr{OS␣is␣exploited}")
for(n in 2:18)
{
lines(p_exp[n,],lwd=2,col=n)
}
abline(h=0,lwd=4)

#now make the probability at OS=k and unexploited
#now the big comparison
plot(freq_os[,90],type="l",lty=3,lwd=4, ylab="Frequency",
xlab="Operating␣System␣at␣time␣t",ylim=c(0,0.35))
lines(freq_os[,90]*(1-p_exp[,90]),lwd=4,lty=1)
lines(freq_os[,190],lwd=4,col=4,lty=3)
lines(freq_os[,190]*(1-p_exp[,190]),lwd=4,col=4,lty=1)
lines(freq_os[,290],lwd=4,col=6,lty=3)
lines(freq_os[,290]*(1-p_exp[,290]),lwd=4,col=6,lty=1)
legend(0.5, 0.35, legend=c("t=90", "t=190","t=290"),
       col=c(1,4,6), lty=c(1,1,1), lwd=3, cex=0.5)
legend(4.75,0.35,legend=c("With␣exploitation","Without␣exploitation"),
lty=c(1,3),cex=0.5)
```
